# Diseases of the Heart and Circulation

**Published:** 1904-07-30

**Authors:** 


### DISEASES OF THE HEART AND CIRCULATION.

(*Concluded from page* 296.)

**Sudden Heart-failure in Toxæmic Conditions.**—Stanley,[4] who has had a large amount of experience both of diphtheria and beri-beri, says it is in these two diseases, *par excellence*, that degeneration of the heart muscle occurs and may lead to sudden death. In both diseases the occurrence of vomiting is almost the invariable herald of a rapidly fatal termination; in addition there occur restlessness, lilac-tinted pallor, increasing weakness of the pulse, and coldness of the extremities. These signs are accompanied and preceded by physical signs of heart muscle weakness—lowered pulse tension, feeble short pulse wave, feeble heart impulse, short first sound, and relatively accentuated pulmonary second sound. In addition the long pause is shortened up to a tic-tac rhythm. This heart failure may occur prior to any manifestation of disturbed innervation, before any paralysis has occurred; about the end of the first week in diphtheria, towards the end of the first month in beri-beri. It is, therefore, not due to any neuritis of the vagus, but is the result probably of direct action of the toxin. The cardiac muscle-fibres undergo a granular albuminous degeneration. The suddenness of the heart failure does not indicate a sudden lesion, but rather is the result of a gradually increasing weakness from muscle degeneration, which may be precipitated by any sudden exertion, but which is the result of the principle of "all or nothing," the transition from "all" (*i.e.* beats sufficient to maintain the circulation) to "nothing" being necessarily rapid.

**Arterio-sclerosis.**—Saville[5] states that three forms of arterio-sclerosis may be recognised—intimal, medial, and adventitial. Atheroma—a patchy fibro-cellular infiltration of the intima—must be regarded as quite distinct from these. The changes in the muscular coat are of chief interest, and the other forms are of no serious import if the media is relatively intact. The morbid changes which may be met with in the media are atrophy, cloudy swelling, granular degeneration, necrosis, calcification, and hypertrophy. This condition of hypermyotrophy is very often antecedent to medial degeneration. It constitutes a distinct pathological condition of prolonged course, capable of recognition during life, sometimes though not necessarily associated with chronic renal disease, and constitutes one of the main pathological conditions on which cerebral and other hæmorrhages depend. The media is increased in thickness, and the heart hypertrophied. In the early stage this is all that is found; in later stages intimal and adventitial sclerosis and degenerations are found. The term "chronic Bright's disease" should not be applied to this generalised condition, consisting of high tension and various circulatory and nutritional defects with fibrosis of organs, unless there are definite evidences of chronic renal disease during life and after death. Stengel[6] says that the etiological factors of arterio-sclerosis are continued mental anxiety, the toxic effect of typhoid fever, alcoholic intemperance, over-feeding, chronic lead-poisoning, and syphilis. The first effects of loss of elasticity in the blood-vessels are an impediment to the easy circulation of the blood and a constant high blood-pressure. Prolongation of the first cardiac and an accentuation of the second sound are present. The earliest symptoms are a failure of vigour, anæmic pallor, and undue sweating on exertion. Active members of the better class are apt to fall early victims to this disease, the easy-going and indolent seldom, except as the result of excesses in eating and drinking. Slight and occasional albuminuria shows that the disease has affected the kidneys, but casts are not found until the disease is advanced. Early cardiac symptoms, such as arrhythmia, increased force of the cardiac impulse, and occasional slight attacks of dyspnœa are frequently encountered. The abuse of tea, coffee and tobacco tends to accelerate the progress of the disease. Allbutt recognises three types of arterio-sclerosis—(1) toxic, due to lead, syphilis, diabetes, or infectious disease; (2) involutionary or senile decay; and (3) secondary to hypertrophy of the muscular coat. The forms 1 and 2 are not necessarily attended by a rise of arterial pressure, but form 3 depends on such a rise of pressure, which is probably the first factor, and in its turn is the result of some alteration in the blood. The blood is either more viscous or has some irritating quality. The production of the condition depends on a repletion, either relative or positive. Weber thinks that arterial hypermyotrophy bears the same relation to arterio-sclerosis that cardiac hypermyotrophy does to cardio-sclerosis. In the majority of cases the hypertrophy of the arterial wall serves a useful purpose. Apparent exceptions are, however, to be found in cardiac or arterial hypermyotrophy of nervous origin. The former, for instance, is present in Grave's disease, the latter in limbs affected by erythromelalgia and Raynaud's disease. These exceptions are probably due to the portions of the nervous system concerned in the reactions in question being themselves abnormal.

[4] Brit. Med. Jour., Dec. 26. [5] Brit. Med. Jour., Feb. 20. [6] Lancet, Jan. 30.

